# Salvianolic Acid A Has Anti-Osteoarthritis Effect *In Vitro* and *In Vivo*

**DOI:** 10.3389/fphar.2020.00682

**Published:** 2020-06-03

**Authors:** Yifan Wu, Zhanghong Wang, Zeng Lin, Xin Fu, Jingdi Zhan, Kehe Yu

**Affiliations:** ^1^Department of Orthopaedics, The Second Affiliated Hospital and Yuying Children’s Hospital of Wenzhou Medical University, Wenzhou, China; ^2^Department of Anesthesiology, The Second Affiliated Hospital and Yuying Children’s Hospital of Wenzhou Medical University, Wenzhou, China

**Keywords:** salvianolic acid A, chondrocytes, osteoarthritis, inflammatory, apoptosis, MAPK, NF-κB

## Abstract

Osteoarthritis (OA) is a degenerative disease found in middle-aged and elderly people, which seriously affects their quality of life. The anti-inflammatory and anti-apoptosis pharmacological effects of salvianolic acid A (SAA) have been shown in many studies. In this study, we intended to explore the anti-inflammatory and anti-apoptotic effects of SAA in OA. We evaluated the expression of pro-inflammatory mediators and cartilage matrix catabolic enzymes in chondrocytes by ELISA, Griess reaction, immunofluorescence, and Western blot, which includes nitric oxide (NO), tumor necrosis factor-alpha (TNF-α), interleukin-6 (IL-6), prostaglandin E2 (PGE2), inducible nitric oxide synthase (iNOS), cyclooxygenase-2 (COX-2), MMPs (MMP-3, MMP-13), and ADAMTS-5. Bax, Bcl-2, and cleaved caspase-3 were also measured by Western blot methods. The results of this experiment *in vitro* showed that SAA not only inhibited the production of inflammatory mediators induced by IL-1β and the loss of cartilage matrix but also reduced the apoptosis of mouse chondrocytes induced by IL-1β. According to the results of immunofluorescence and Western blot, SAA inhibited the activation of the NF-κB pathway and MAPK pathway. The results of these *in vitro* experiments revealed for the first time that SAA down-regulated the production of inflammatory mediators and inhibited the apoptosis of mouse chondrocytes and the degradation of extracellular matrix (ECM), which may be attributed to the inhibition of the activation of NF-κB and MAPK signaling pathways. In the *in vivo* experiments, 45 mice were randomly divided among three groups (the sham group, OA group, and OA + SAA group). The results of animal experiments showed that SAA treatment for eight consecutive weeks inhibited further deterioration of OA. These results demonstrate that SAA plays an active therapeutic role in the development of OA.

## Introduction

Osteoarthritis (OA) is a joint degenerative disease that seriously affects the quality of life of patients, leading to reduced function and even disability, which brings a huge economic burden to patients, families, and society (Kon et al., 2012; Johnson and Hunter, 2014). OA tends to occur in the middle-aged and elderly population, and the incidence of OA is increasing in countries where population aging is progressing (Lee and Kim, 2017; Prashansanie Hettihewa and Gunawardena, 2018; Rezus et al., 2019). It was reported that OA not only led to joint pain, deformity, and dyskinesia but also increased the incidence of cardiovascular disease (Fernandes and Valdes, 2015). At present, more and more scholars have been investigating the molecular mechanism of OA (Lieberthal et al., 2015; Marchev et al., 2017). Therefore, it’s particularly important to further study and improve the pathogenesis of OA to prevent the progress of OA.

Normal articular cartilage contains many cartilage extracellular matrixes, which are mainly composed of collagen (mostly type II collagen) and proteoglycan. These are very important to the biomechanical properties of cartilage and provide key elastic supports for joint activities (Wieland et al., 2005; Sarin et al., 2019). Cartilage is the most important pathological tissue of OA. Chondrocytes are the only cellular components in tissues that are involved in joint degeneration. The main pathological changes are the gradual destruction of the extracellular matrix (ECM) and the apoptosis of chondrocytes (Thomas et al., 2007). At present, the pathogenesis of OA is not clear, but more and more studies showed that the development of OA was closely related to the secretion of cytokines. It’s noteworthy that IL-1β played an important role in the entire process of chondrocyte inflammation and apoptosis (Chen et al., 2018; Sun and Hu, 2019). During the pathogenesis of OA, the increase of IL-1β not only results in the production of pro-inflammatory factors, including matrix metalloproteinases (MMPs), thrombin sensitive protein motifs (ADAMTS), inducible nitric oxide synthase (iNOS), cyclooxygenase-2 (COX-2), and prostaglandin E2 (PGE2), but also increases the expression of apoptosis-promoting factors (such as Bax and cleaved caspase-3) (Kim et al., 2000; Sharif et al., 2004; Smith, 2006; Echtermeyer et al., 2009; Cheng et al., 2011). A number of studies have shown that the expression of the IL-1β-induced pro-inflammatory factors was achieved through the NF-κB pathway. IL-1β led to the degradation of IκBα after phosphorylation and promoted the entry of nuclear factor NF-κB into the nucleus, which mediated the secretion of inflammatory factors in chondrocytes (Marcu et al., 2010; Hayden and Ghosh, 2011). Besides, the NF-κB signaling pathway induced by IL-1β was not only involved in the inflammatory reaction against chondrocytes but also involved in the apoptosis of chondrocytes (Zhang et al., 2015). The signal pathway of the mitogen-activated protein kinase (MAPK) family mainly includes extracellular signal-regulated kinase (ERK), c-Jun N-terminal kinase (JNK), and p38 (Geng et al., 1996; Johnson and Lapadat, 2002). Previous studies have shown that the activation of JNK and p38-MAPK promoted inflammation and apoptosis, and the ERK signaling pathway was also essential for cell survival (Iwayama et al., 2011). In addition, there is evidence that the use of MAPK inhibitors helps to reduce the expression of IL-1β-induced MMPs and alleviate the extracellular matrix (ECM) degradation (Liacini et al., 2002; Xie et al., 2015). Therefore, inhibiting these inflammatory pathways and reducing apoptosis of inflammation-related chondrocytes were considered to be an effective method for the treatment of OA.

Salvianolic acid A (SAA) extracted from the roots of *Salvia miltiorrhiza Bunge* (an edible food), has been shown to have anti-inflammatory and anti-apoptosis effects in many diseases (Su et al., 2015). Previous studies have found that SAA could protect from myocardial ischemia/reperfusion injury by reducing the production of inflammatory mediators (Yuan et al., 2017). Treatment of SAA exerts myocardial protection through activating Trx and inhibiting the JNK signaling pathway (Zhou et al., 2019). Some scholars have found in animal experiments that SAA impairing NF-κB signaling and increasing the expression of Bcl-2 has an anti-apoptosis function on damaged brain tissues (Chien et al., 2016). SAA binds to Toll-like receptor 4 (TLR4) to prevent the potential effects of LPS-challenged acute kidney injury (Zeng et al., 2020). In addition, SAA decreased production of TNF-α, IL-6, iNOS, and COX-2 by inhibiting the activation of NF-κB and MAPK signal pathways in HK-2 cells (Huang et al., 2013; Zhang et al., 2018). However, the role of SAA in the development of OA through the MAPK and NF-κB signaling pathways needs further exploration. In the present work, we speculated that SAA may alleviate the progress of OA by inhibiting inflammation and related apoptosis and that MAPK and NF-κB signaling pathways may be involved in this process.

## Materials and Methods

### Reagents

Salvianolic acid A (98% in purity) was obtained from Solarbio (Beijing, China), and Fetal bovine serum (FBS) and Dulbecco’s modified Eagle’s Medium F12 (DMEM/F12) were purchased from Invitrogen (Carlsbad, CA, USA). Dimethylsulfoxide (DMSO) was purchased from Sigma Aldrich (St Louis, MO, USA), and SAA was dissolved in DMSO and stored at 4°C. Cell counting kit-8 and caspase-3 cellular activity assay kit were purchased from Beyotime (ShangHai, China). IL-1β was obtained from PeproTech Inc. (NJ, USA). Primary antibodies against collagenase type II, COX-2, iNOS, MMP-3, ADAMTS-5, Lamin B1, and GAPDH were purchased from Proteintech Group (WuHan, China) and JNK, p-JNK, ERK, p-ERK, p38, and p-p38 were obtained from CST (MA, USA). Antibodies against p65 and IκBα were purchased from Cell Signaling Technology Sigma Aldrich (St Louis, MO, USA). Cleaved caspase3, Bax, and Bcl-2 antibodies were obtained from Wanleibio (Shenyang, China). Enzyme-linked immunosorbent assay (ELISA) kits to detect prostaglandin E2 (PGE2) were purchased from R&D Systems (Minneapolis, MN, USA). The Mouse MMP-3 ELISA kit was obtained from Solarbio Life Science (Beijing, China). Griess reagents were obtained from Bio-Swamp Life Science (Shanghai, China). Goat anti-rabbit and anti-mouse IgG-HRP were supplied by Boster (Wuhan, China), and Alexa Fluor^®^488-labeled and Alexa Fluor^®^594-labeled goat anti-rabbit IgG (H+L) secondary antibody were purchased from Jackson ImmunoResearch (West Grove, PA, USA). The 4’,6-diamidino-2-phenylindole (DAPI) was purchased from Beyotime (Shanghai, China).

### Primary Mice Chondrocyte Culture

Ten C57BL/6 mice (5 males and 5 females, 10-days-old) were sacrificed following ethical approval obtained from the Medical Ethical Committee of the Second Affiliated Hospital, Wenzhou Medical University, and following the guidelines of the Animal Care and Use Committee of Wenzhou Medical University. After euthanization of mice (Animal Center of Chinese Academy of Sciences, Shanghai, China), articular cartilage was obtained from the knee joint and hip joint of the mice. Firstly, the articular cartilage pieces of the knee and hip joint of mice were washed with PBS two to three times. Before incubation with 2 mg/ml (0.1%) collagenase II for 4 to 6 h at 37°C, digestion was performed with 5 ml 0.2% type II collagenase in 0.2% trypsin-EDTA solution for 45 min. The digested cartilage tissue was added to the centrifuge tube of 15 ml and then centrifuged at 1,000 rpm for 3 min at 37°C. Then, the sediment was re-suspended with cell culture medium, which contained DMEM/F-12(89%), FBS(10%), penicillin and streptomycin antibiotics (1%). And cell pellets were implanted into 100 mm culture flasks and then incubated in 5% CO_2_ at 37°C. Until the confluence rate of cartilage cells reached 80% ~ 90%, 0.25% trypsin-EDTA (Djibouti, Invitrogen) was used to harvest the cells after chondrocyte were treated with or without SAA and IL-1β. Then, at the appropriate concentration, these cells were seeded into 100 mm culture flasks. The isolated primary chondrocytes were transferred to cryotubes and placed in a refrigerator at -80°C for subsequent cell experiments. Only chondrocytes of the 1st to 3rd generations were used in the experiment to avoid phenotypic changes.

### Effect of SAA on Chondrocyte Viability

After treatment, cell viability was assessed by a Cell Counting Kit-8 (CCK-8; Beyotime, ShangHai, China) according to the manufacturer’s instructions. Mouse chondrocytes were seeded in a 96-well plate at a density of 5 × 10^3^ cells per well in 100 µL of DMEM/F-12 medium and incubated at 37°C. The cells were then pretreated with increasing concentrations (0, 6.25, 12.5, 25, 50, 100, and 200 μM) of SAA for either 24 h or 48 h. Next, 10 μl of CCK-8 solution was added in each well. Mouse chondrocytes were incubated for another 2 h at 37°C before optical density was calculated by spectrophotometer. All experiments were performed five times.

### Caspase-3 Activity Test

The mouse chondrocytes were pretreated with SAA at various concentrations (0, 6.25, 12.5, and 25 μM) for 24 h with or without rat recombinant IL-1β (10 ng/ml) for another 24 h. After collecting and lysing chondrocytes, caspase-3 activity was determined immediately. The activity of caspase-3 in five groups of cartilage cells was detected by caspase-3 cellular activity assay kit (Beyotime, ShangHai, China) according to the manufacturer’s operating guidelines. In short, 40 μl of detection buffer, 50 μl of sample or lysate were sequentially added to the 96-well plate, followed by 10 μl of Ac-DEVD-pNA (2 mM) and divided into five groups with a similar treatment. Next, it was incubated at 37°C for 60-120 min. Finally, the absorbance of pNA catalyzed by caspase-3 in different samples was determined by using a microplate system at 405 nm. All experiments were performed five times.

### ELISA and the Griess Reaction

Mouse chondrocytes were seeded in 6-well plates and exposed to different concentrations (0, 6.25, 12.5, and 25 μM) of SAA 24 h prior to treatment with IL-1β (10 ng/ml). The cells cultured for another 24 h, and then the Griess reagent used as previously described was used to measure the concentration of NO (Au et al., 2007). By using a spectrophotometer to take a measurement, the optical density of each sample was obtained. Besides, ELISA kits were used to measure the concentration of PGE2 and MMP-3 following the manufacturer’s guidelines. All experiments were performed five times.

### Western Blot Analysis

Equal amounts of protein (40 ng) extracted from chondrocytes were separated by 10% sodium dodecyl sulfate polyacrylamide gel electrophoresis (SDS-PAGE) gels and then transferred to polyvinylidene fluoride (PVDF) membranes (Bio-Rad, USA) at a constant current (300 mA) for 1 to 2 h. The membrane was blocked with 5% skim milk for 2 h before incubation with the primary antibodies against collagenase type II (1: 1000), COX-2 (1: 1000), iNOS (1: 1000), MMP-3 (1: 1000), ADAMTS-5 (1: 1000), Lamin B1 (1: 1000), GAPDH (1: 1000), JNK (1: 1000), p-JNK (1: 1000), ERK (1: 1000), p-ERK (1: 1000), p38 (1: 1000), p-p38 (1: 1000), p65 (1: 1000), IκBα (1: 1000), Cleaved caspase-3 (1: 1000), Bax (1: 1000), and Bcl-2 (1: 1000) overnight at 4°C. Subsequently, the membranes were incubated with the corresponding secondary antibodies for 2 h at room temperature. Eventually, the blots were visualized by Enhanced Chemiluminescence (ECL) kit, and the intensities of these blots were quantified using Image Lab 3.0 software (Bio-Rad). All experiments were performed five times.

### Immunofluorescence

Chondrocytes were seeded in 6-well plates and were treated with or without SAA (25 μM) for 24 h, and then stimulated with or without IL-1β (10 ng/ml) for another 24 h. PBS was used to wash the cells three times and then treated with 4% paraformaldehyde for 15 min. After fixation, the chondrocytes were rinsed three times. Next, cells were treated with 0.1% Triton X-100 at room temperature for another 15 min. Blocked with goat serum for 1 h, mouse chondrocytes were incubated overnight with the dilution of the primary antibodies (MMP-13 (1:200) and p65 (1: 200)) at 4°C. After washing with PBS, chondrocytes were incubated with Alexa Fluor^®^488-labeled and Alexa Fluor^®^594-labeled goat anti-rabbit IgG (H+L) secondary antibody (1:400) in darkness for 1 h. Finally, they were washed three times with PBS before and after incubation with 4,6-diamino -2- phenylindole (DAPI) for 10 min. We randomly captured five areas of each slide from different groups (the control group, the IL-1β group, and the IL-1β + SAA group) using a fluorescence microscope (Olympus Inc., Tokyo, Japan). Finally, observers who were not involved in the experiment evaluated the fluorescence intensity using the Image J software 2.1 (Bethesda, MDUSA). Five areas were randomly selected for observation with a fluorescence microscope (Olympus Inc., Tokyo, Japan). The calculation of the average fluorescence intensity was done by professional researchers who were blinded to the experiment. All experiments were performed five times.

### TUNEL Method

The terminal deoxynucleotidyltransferase (TdT) dUTP nick end labelling (TUNEL) method was used to measure apoptotic DNA fragmentations of chondrocytes. Chondrocytes seeded in 6-well plates were treated with or without SAA (25 μM) for 24 h, and then stimulated with or without IL-1β (10 ng/ml) for another 24 h. PBS was used to wash the cells three times and then treated with 4% paraformaldehyde for 15 min. After fixation, chondrocytes were permeabilized with 0.2% Triton X-100 for 10 min at room temperature. Next, mouse chondrocytes were washed with PBS three times and then stained *in situ* using a cell death detection kit (Hoffmann-La Roche Ltd., Basel, Switzerland) according to the manufacturer’s instructions. After chondrocytes were stained with 40, 6-diamidino-2-phenylindole (DAPI), a Nikon ECLIPSE Ti microscope was used to observe apoptotic chondrocytes in each group. Finally, five areas were randomly selected for observation with a confocal laser scanning microscope (Leica Microsystems, Germany). The rate of TUNEL-positive cells in each field was calculated. All experiments were performed five times.

### Establishment of the Mice OA Model

Forty-five 10-week-old C57BL/6 male wild-type (WT) mice were bought from the Animal Center of the Chinese Academy of Sciences (Shanghai, China). The animal experiment was authorized by the Animal Care and Use Committee of Wenzhou Medical University. Mice were randomly allocated to three groups (n = 15 mice per group): (1) the sham group, (2) OA group (received intraperitoneal injections of DMSO), and (3) OA +SAA group (received SAA dissolved in DMSO by intraperitoneal injections in equal doses). An OA model was made by surgical destabilization of the medial meniscus (DMM) as previously described (Vasheghani et al., 2015). In brief, under the anesthesia of mice, the right knee joint capsule was cut from the medial patellar tendon of the OA group and OA +SAA group mice, and the medial meniscus ligament was cut with microsurgical scissors, while the mice in the sham group only cut the joint capsule without damaging the medial meniscus ligament. Mice were sacrificed at the eighth week after surgery, and the knee joints of mice were collected for further histopathological analysis.

### Histological Analysis

Firstly, 4% paraformaldehyde was used to fix the knee joint for 48 h at room temperature. The knee joint was decalcified in 10% EDTA solution at room temperature for 4 weeks, embedded in paraﬃn blocks, and cut into 6μm thick slices. Secondly, knee sections from different groups (the sham group, OA group, and OA + SAA group) were stained with Modified Safranine O-Fast Green FCF Cartilage Stain (S-O) Kit which was purchased from Solarbio (Beijing, China). 15 mice in each group (the sham group, OA group, and OA + SAA group) were used for histomorphometric scoring. The sliced pigmented knee joints were observed under a microscope by blind method, and the degree of articular cartilage damage was assessed using the Osteoarthritis Research Society International (OARSI) scoring system as previously described (Gerwin et al., 2010). According to the previous description (Permuy et al., 2015), we measured the thickness of the medial subchondral bone plate from the Safranin O stained sections *via* Axio Vision software. Cartilage evaluation was mainly done by researchers who did not participate in animal experiments.

### Statistical Analysis

All *in vitro* experiments were performed five times, while *in vivo* studies were performed once. In cell culture experiments, ten C57BL/6 mice (5 males and 5 females, 10 days old) were sacrificed and used to extract chondrocytes. Through the CCK-8 experiment, we found that SAA has no toxic effect on chondrocytes in the range of 0-25 μM. In the Caspase-3 activity test, ELISA, Griess Reaction, and Western blot experiments, we divided the mice into five groups as follows: control, IL-1β, IL-1β + SAA (6.25 μM), IL-1β + SAA (12.5 μM), and IL-1β + SAA (25 μM). In the Immunofluorescence and TUNEL experiment, we divided the mice into three groups as follows: control, IL-1β, and IL-1β + SAA (25 μM). Finally, data was collected and statistically analyzed using SPSS statistical software version 16.0. In animal experiments, forty-five 10-week-old C57BL/6 male wild-type (WT) mice were randomly allocated to three groups (n = 15 mice per group): (1) the sham group, (2) OA group, and (3) OA +SAA group. We collected the knee joints of mice in each group and made them into 6μm thick slices and stained them with S-O staining. Then, we compared the thickness of the subchondral bone plate and the OARSI score to assess the degree of articular cartilage degradation. Finally, we used SPSS statistical software version 16.0 for statistical analysis.

The data of the experimental results are expressed by the mean ± standard deviation (SD), and then the statistical analysis was carried out by using the SPSS statistical software version 16.0. In this study, one-way analysis of variance (ANOVA) was used to compare multiple factors and verified the assumption of normality and tested the homogeneity of variance. Data were analyzed by one-way analysis of variance (ANOVA) followed by Tukey’s test for statistical comparisons. The data differences between the groups were further analyzed and the average difference was expressed as a 95% confidence interval (CI). Nonparametric data (like OARSI score) were analyzed by the Kruskal-Wallis H test. P < 0.05 was regarded as having statistical significance. The following symbols were used to indicate statistical significance: * indicate P < 0.05, ** and ## indicate P < 0.01.

## Results

### Effect of SAA on the Toxicity of Cartilage Cells *In Vitro*

**Figure 1:**
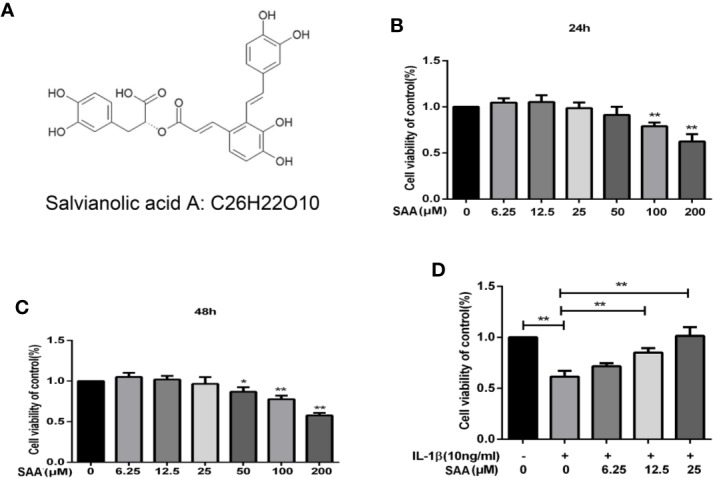
Pharmacological effect of SAA on mouse chondrocytes cultured *in vitro*. **(A)** Chemical structure of SAA. **(B, C)** The inhibitory effect of SAA on the growth of mouse chondrocytes at different concentrations (0, 6.25, 12.5, 25, 50, 100, and 200 µM) was determined by CCK-8 assay at 24 and 48 h. **(D)** The CCK-8 results of mouse chondrocytes induced by IL-1β (10 ng/ml) were observed 24 h after pre-treatment with SAA (0, 6.25, 12.5, and 25 µM). The data obtained in this picture are represented by mean ± standard deviation. In **(A–C)**, *P < 0.05 and **P < 0.01, compared with the control group. In **(D)**, significant differences between the two groups are expressed as **P < 0.01, n = 5 independent experiments.

**Figure 1A** shows the molecular chemical structure of SAA. To ensure the safety of SAA on mouse chondrocytes, CCK-8 assay was used to detect the toxic effect of SAA on mouse chondrocytes. We observed the cytotoxic effects of SAA (0, 6.25, 12.5, 25, 50, 100, and 200 μM) at different concentrations on chondrocytes after 24 h and 48 h. As shown in **Figures 1B, C**, the results of the CCK-8 assay showed that SAA at 6.25, 12.5, and 25 μM had no obvious cytotoxicity effect on cultured mouse chondrocytes. Compared with untreated mouse chondrocytes, the higher concentration of SAA (50, 100, and 200 μM) reduced viability of chondrocytes. Therefore, SAA at a concentration of 0–25 μM were considered to be nontoxic concentrations and allowed to be used in subsequent experiments. From **Figure 1D**, we found that SAA reversed the cell viability treated with IL-1β.

### Effect of SAA on the Expression of Inflammatory Mediators in IL-1β-Induced Mouse Chondrocytes

**Figure 2:**
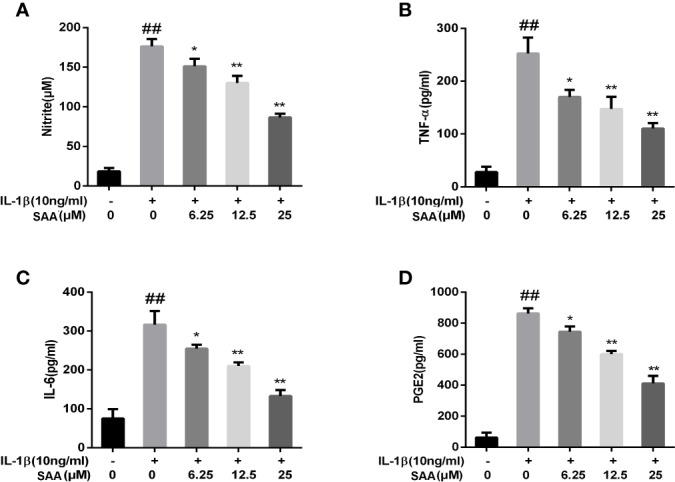
Effect of SAA pretreatment on the expression of inflammatory mediators (NO, TNF-α, IL-6, and PGE2) induced by IL-1β in mouse cartilage cells. **(A)** Determination of the content of nitrite in each group by Griess reaction. **(B–D)** ELISA was used to assess the concentration of TNF-α, IL-6, and PGE2 in each group. The data obtained in this picture are represented by mean ± standard deviation. *^##^P* < 0.01, vs. control group; *P < 0.05, **P < 0.01, vs. group induced by IL-1β alone, n = 5 independent experiments.

**Figure 3:**
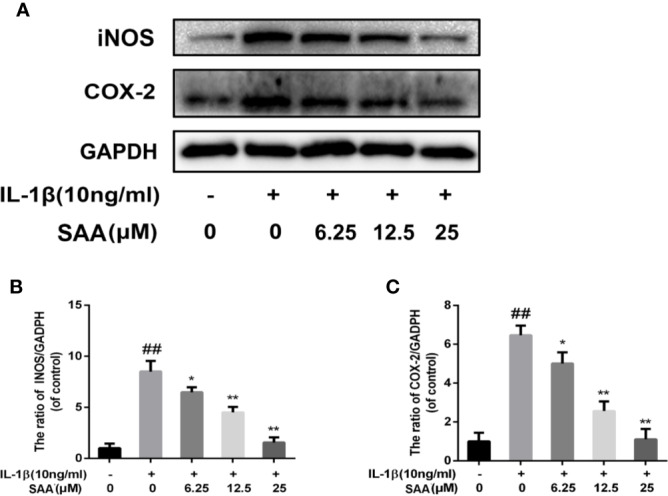
Effects of different concentrations of SAA on the expression of iNOS and COX-2 in mouse chondrocytes induced by IL-1β. After mouse chondrocytes pretreated with SAA (0, 6.25, 12.5, and 25 µM) were treated with IL-1β (10 ng/ml) for 24 h, **(A–C)** Western blot analysis was used to analyze protein expression levels of iNOS and COX-2. The data obtained in this picture are represented by mean ± standard deviation. *^##^P* < 0.01, vs. control group; *P < 0.05, **P < 0.01, vs. group induced by IL-1β alone, n = 5 independent experiments.

Next, to investigate whether SAA inhibited IL-1β-induced inflammation, Griess reaction, Western blot, and ELISA were used to detect the expression levels of NO, PGE2, TNF-α, IL-6, iNOS, and COX-2, respectively. PGE2, TNF-α, and IL-6 concentrations in the culture supernatant of mouse chondrocytes were measured by ELISA kits, while NO levels were measured using the Griess reaction. According to the results of the ELISA kits and Griess reaction (**Figures 2A–D**), in the IL-1β group, the expression of PGE2, TNF-α, and IL-6 and the production of NO were significantly higher than those in the untreated group, however, pretreatment with SAA reversed IL-1β-induced PGE2, TNF-α, IL-6, and NO production in a dose-dependent manner. Moreover, the results of Western blot (**Figures 3A–C**) showed that IL-1β (10 ng/ml) stimulation alone obviously increased the expression of inflammatory mediators (COX-2, iNOS) at the protein level, while mouse cartilage cells pretreated with SAA (12.5, 25 μM) for 24 h evidently down-regulated the expression of iNOS and COX-2.

### SAA Plays a Protective Role in the ECM Degradation of Mouse Chondrocytes Induced by IL-1β

**Figure 4:**
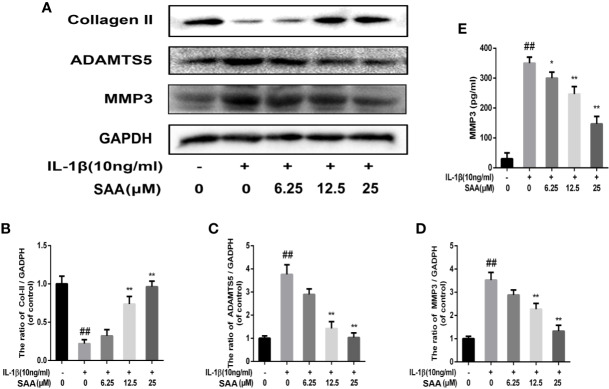
Effect of SAA on ECM degradation of mouse chondrocytes. After mouse chondrocytes pretreated with SAA (0, 6.25, 12.5, and 25 µM) were treated with IL-1β (10 ng/ml) for 24 h, **(A–D)** Western blot was used to analyze the expression of type II collagen, ADAMTS-5, and MMP-3 protein. **(E)** ELISA was used to assess the concentration of MMPP-3 in each group. The data obtained in this picture are represented by mean ± standard deviation. *^##^P* < 0.01, vs. control group; *P < 0.05, **P < 0.01, vs. group induced by IL-1β alone, n = 5 independent experiments.

**Figure 5:**
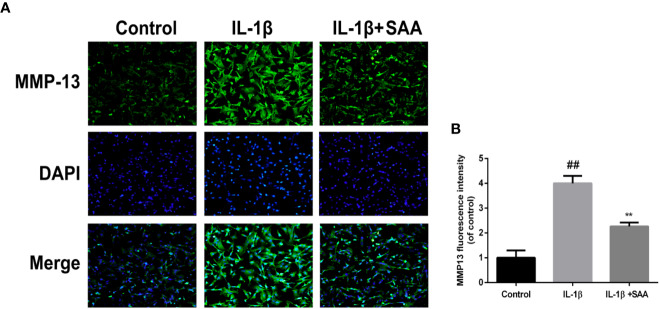
Immunofluorescence staining of the MMP-13 protein in mouse chondrocytes. **(A)** The expression of the MMP-13 protein (green) in the cytoplasm was analyzed by immunofluorescence after mouse chondrocytes pretreated with SAA (25 µM) were treated with IL-1β (10 ng/ml) for 24 h. **(B)** The fluorescence intensities of MMP-13 were determined using Image J software. The experiment was repeated three times. The data obtained in this picture are represented by mean ± standard deviation. *^##^P* < 0.01, vs. control group; **P < 0.01, vs. group induced by IL-1β alone, n=5 independent experiments.

The effects of SAA on the metabolic activity of ECM in mouse chondrocytes were detected by Western blot and immunofluorescence. In the first place, we observed the effect of SAA on the expression of collagenase type II in mouse chondrocytes induced by IL-1β (10 ng/ml). As shown in **Figures 4A, B**, compared with the untreated cells, the production of type II collagen was significantly down-regulated after stimulation with IL-1β (10 ng/ml). However, it was worth noting that this IL-1β-induced down-regulation trend was reversed by SAA in a dose-dependent manner. Moreover, according to the results of Western blot (**Figures 4A, C, D**), we found that SAA inhibited the up-regulation of MMP-3 and ADAMTS-5 protein expression in mouse chondrocytes induced by IL-1β (10 ng/ml). As shown in **Figure 4E**, the inhibitory effect of SAA on MMP-3 expression in a concentration gradient was observed by ELISA. The results of the immunofluorescence staining showed that SAA inhibited the significant expression of MMP-13 in the cytoplasm (**Figures 5A, B**).

### The Treatment of SAA Effectively Inhibits the Apoptosis of Mouse Chondrocytes

**Figure 6:**
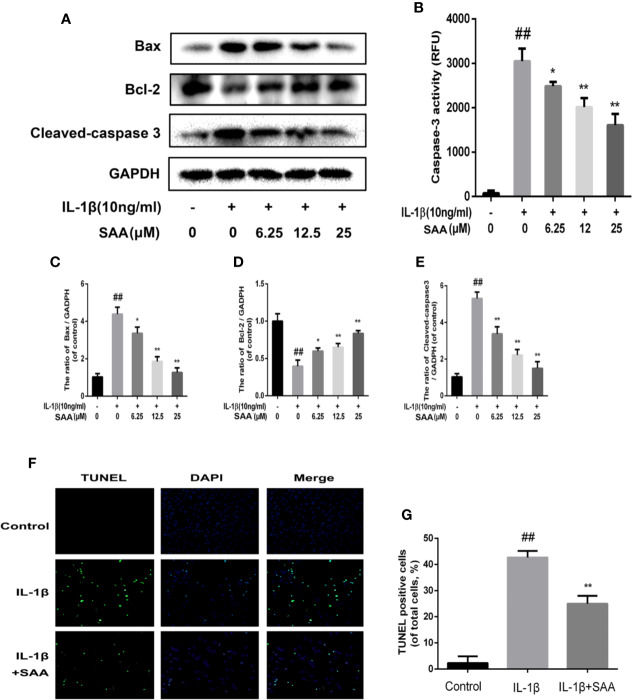
Anti-apoptosis effect of SAA on mice. Mouse chondrocytes pretreated with SAA (0, 6.25, 12.5, and 25 µM) were treated with IL-1β (10 ng/ml) for 24 h. **(B)** The activity of caspase 3 was measured by a caspase 3 cell activity assay kit. **(A, C–E)** The expression levels of anti-apoptotic protein (Bcl-2) and apoptotic protein (Bax and cleaved caspase-3) were detected by Western blot analysis. **(F, G)** The number of apoptotic chondrocytes was determined using TUNEL staining and quantified. The data obtained in this picture are represented by mean ± standard deviation. *^##^P* < 0.01, vs. control group; *P< 0.05, **P< 0.01, vs. group induced by IL-1β alone, n = 5 independent experiments.

To detect whether SAA could inhibit the apoptosis of mouse chondrocytes induced by IL-1β (10 ng/ml), TUNEL staining and caspase-3 cellular activity assay kit were used to evaluate the apoptotic changes of chondrocytes. As shown in **Figure 6B**, we found that caspase-3 activity was increased under IL-1β stimulation, while the activity of caspase-3 in mouse chondrocytes decreased after pretreatment with SAA for 24 h. Bax and cleaved caspase-3 leading to chondrocyte apoptosis were significantly up-regulated by IL-1β. IL-1β decreased the expression of Bcl-2, which played an inhibitory role in the apoptosis of mouse chondrocytes. But, after pretreatment with SAA for 24 h, the tendency induced by IL-1β was reversed in a dose-dependent manner (**Figures 6A, C–E**). Most importantly, the results of TUNEL staining are consistent with those of WB. This more strongly illustrates that the treatment of SAA reduces the number of apoptotic chondrocytes (**Figures 6F, G**).

### Effect of SAA on NF-κB Activation of Mouse Chondrocytes

**Figure 7:**
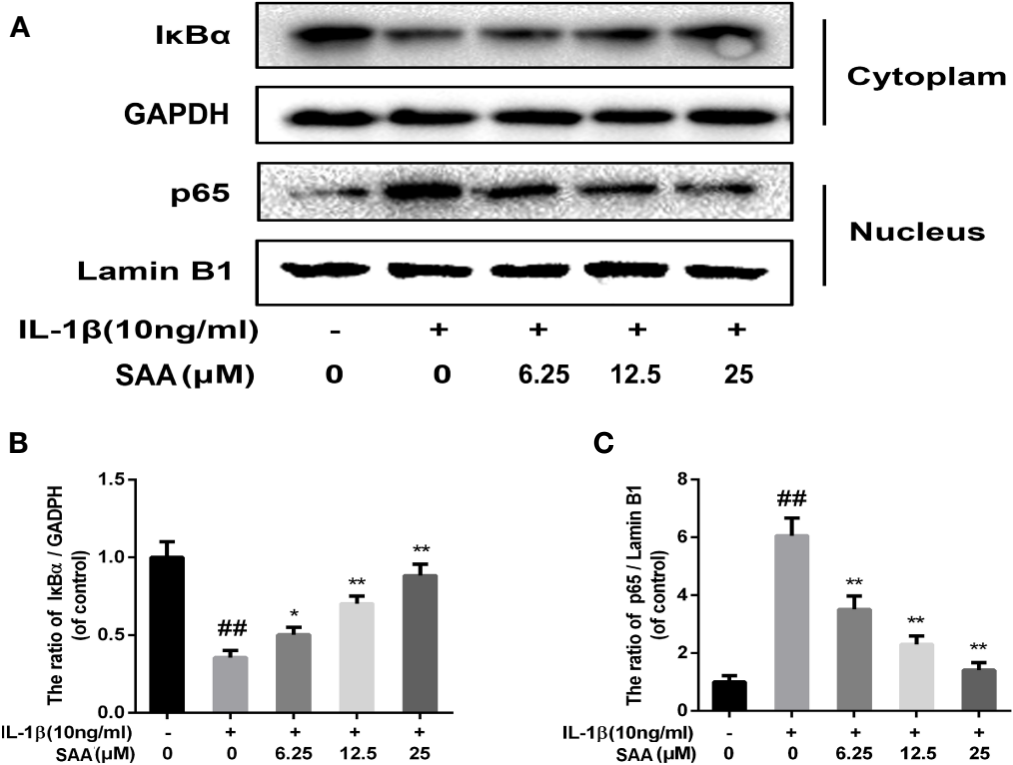
The effect of SAA on the activation of the NF-κB signal pathway induced by IL-1β. Mouse chondrocytes pretreated with SAA (0, 6.25, 12.5, and 25 µM) were treated with IL-1β (10 ng/ml) for 24 h. **(A–C)** The expression levels of IκBα in cytoplasm and p65 in nuclear were detected by Western blot analysis. The data obtained in this picture are represented by mean ± standard deviation. *^##^P* < 0.01, vs. control group; *P < 0.05, **P < 0.01, vs. group induced by IL-1β alone, n = 5 independent experiments.

**Figure 8:**
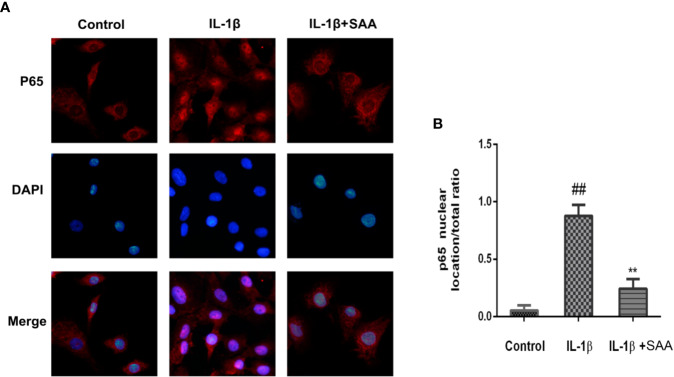
Immunofluorescence staining of P65 protein in mouse chondrocytes. **(A)** Observation of the distribution of p65 proteins in the cytoplasm or nucleus by immunofluorescence staining. **(B)** p65 nuclear location/total ratio is quantitatively analyzed by Image J software. The experiment was repeated three times. The data obtained in this picture are represented by mean ± standard deviation. *^##^P* < 0.01, vs. control group; **P < 0.01, vs. group induced by IL-1β alone, n = 5 independent experiments.

Numerous studies have shown that activation of the NF-κB pathway was associated with the progress of OA. The degradation of IκBα and the nuclear translocation of NF-κB (p65) indicated the activation of NF-κB signaling pathway. To investigate the mechanism of anti-inflammatory and anti-apoptosis effects of SAA, the expression of IkBα in cytoplasm and p65 in the nucleus of mouse cartilage cells were detected by Western blot. From **Figures 7A–C**, we found that mouse chondrocytes stimulated by IL-1β not only outstandingly promoted the degradation of IkBα in the cytoplasm but also induced p65 translocation into the nucleus. On the contrary, pretreatment with SAA reversed IL-1β-induced p65 and IkBα expression in a dose-dependent manner. Immunofluorescence results of p65 showed that the p65-active proteins were mainly localized in the cytoplasm of mouse chondrocytes without IL-1β pretreatment, while p65-active proteins were translocated into the nucleus in the IL-1β treatment group. (**Figure 8A**). However, SAA could greatly block the translocation of p65 from the cytoplasm to the nucleus (**Figures 8A, B**). These results suggested that SAA has a protective effect on mouse OA chondrocytes by inhibiting the activation of the NF-κB pathway.

### Effect of SAA on the MAPK Signaling Pathway

**Figure 9:**
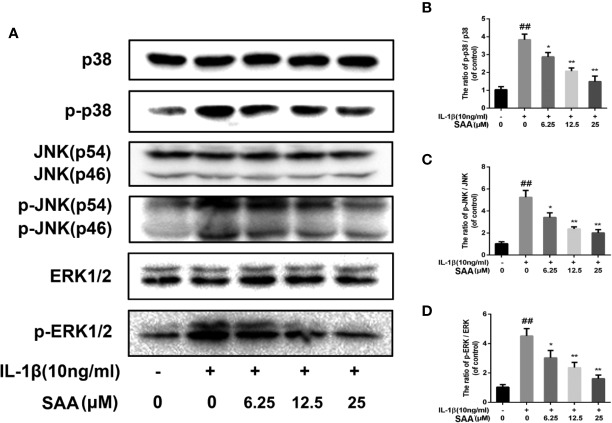
The effect of SAA on the activation of the MAPK signal pathway induced by IL-1β. Mouse chondrocytes pretreated with SAA (0, 6.25, 12.5, and 25 µM) were treated with IL-1β (10 ng/ml) for 24 h. **(A-D)** The expression levels of p38, p-p38, JNK, p-JNK, ERK, and p-ERK were detected by Western blot analysis. The data obtained in this picture are represented by mean ± standard deviation. *^##^P* < 0.01, vs. control group; *P < 0.05, **P < 0.01, vs. group induced by IL-1β alone, n = 5 independent experiments.

Recent studies have demonstrated that, in addition to the NF-κB pathway, the MAPK pathway was also closely related to the occurrence of OA. Therefore, we studied the possible mechanism of SAA in the IL-1β-induced MAPK pathway. As shown in **Figures 9A–D**, compared with the untreated group, p38, JNK, and ERK were significantly phosphorylated under the stimulation of IL-1β. Nevertheless, SAA pretreatment inhibited the activation of p38, JNK, and ERK. It was suggested that the protective effect of SAA on mouse chondrocytes may be related to the inhibition of the activation of the MAPK pathway.

### Experimental Study on the Effect of SAA on Cartilage Degeneration in a Mouse Model of OA

**Figure 10:**
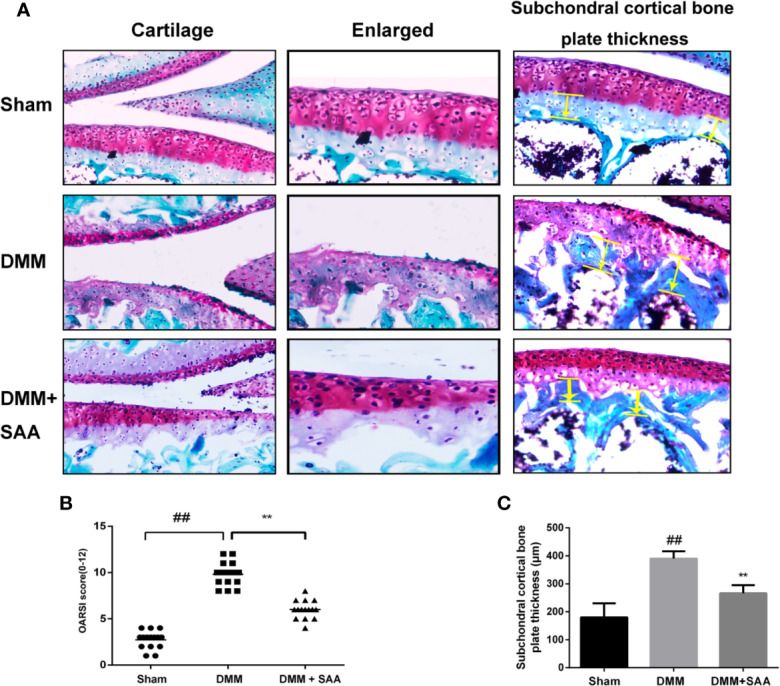
SAA plays a protective role in the deterioration of the mouse model of OA. **(A)** SafraninO staining was used to evaluate the morphological differences of mouse knee joint sections among the three groups (the sham group, the OA group, and the OA + SAA group); the yellow lines indicate the areas of subchondral cortical bone thickness. **(B)** International (OARSI) scores of the three groups. **(C)** Schematic diagram of subchondral cortical bone plate thickness. The data obtained in this picture are represented by mean ± standard deviation. ^##^*P* < 0.01, vs. sham group; **P < 0.01, vs. DMM group, n = 15 mice/group.

We have demonstrated the efficacy of SAA in OA by applying SAA to the mouse OA model. Safranin O staining and OARSI score could be used to analyze the severity of OA. As shown in **Figure 10A**, comparing the results of Safranin O staining between the sham group and OA group, we found that the articular cartilage surface of the OA group showed greater cartilage wear and cartilage thickness reduction, and the difference was statistically significant (p < 0.01). However, after eight weeks of continuous treatment with SAA, mouse models of OA were not only repaired on the cartilage surface of the joint, but also reversed the decrease of cartilage thickness and subchondral cortical bone plate thickness (**Figure 10C**), which were significantly different from that in the OA group (P < 0.01). After a quantitative analysis of the OARSI score (**Figure 10B**), we also found that the *mean* of OARSI scores in the OA + SAA group was between the OA group and sham group, and there was a significant difference between the OA + SAA group and OA group (P < 0.01). The results revealed that in the mouse OA models, the development of OA was distinctly improved under the action of SAA.

## Discussion

As a part of the clinical syndrome of OA, articular cartilage degeneration is one of the causes of progressive pain and disability in many elderly people (Pap and Korb-Pap, 2015). The study found that obesity, old age, and other common factors in daily life will increase the prevalence of OA, so the prevalence of OA is still on the rise (Reijman et al., 2007). So far, no effective treatment for OA has been found (Saxby and Lloyd, 2017). At present, NSAIDs, a non-steroidal anti-inflammatory drug, is mainly used to relieve pain, redness, and swelling caused by OA, but these drugs produce a series of adverse reactions (Kowalski et al., 2011). Studies have also shown that the withdrawal rate of NSAIDs in patients with OA was very high, which was not conducive to the treatment of patients (Yilmaz et al., 2005). As a kind of traditional Chinese medicine, SAA has been found to have a variety of pharmacological effects, like protective effects for the heart and the brain. In this study, both *in vitro* and *in vivo* experiments confirmed that SAA exerted anti-inflammatory and anti-apoptotic effects.

Inflammatory cytokines played a major role in the development of OA. The up-regulation of IL-1β triggered a series of intracellular events, which not only led to the activation of protease and the high expression of inflammatory mediators, such as NO, COX-2, PGE2, and MMPs, but also inhibited the synthesis of ECM (Daheshia and Yao, 2008). Extensive evidence suggested that NO was associated with the pathogenesis of OA. NO was considered to be a decomposable factor, which was involved in the pathological process of OA by inducing apoptosis of chondrocytes, synthesis of MMPs, and expression of pro-inflammatory cytokines (Santoro et al., 2015). INOS was an enzyme responsible for NO production and a disruptive factor involved in the OA process. At the site of inflammation, COX-2 may be released in large quantities from synovial cells, participate in the inflammatory process, and cause cartilage damage (Chowdhury et al., 2006; Huang et al., 2009). Matrix metalloproteinases (MMPs), including MMP-1, MMP-2, MMP-3, MMP-9, and MMP-13, were enzymes that could degrade all components of ECM. MMPs not only degraded collagen, but also degraded proteoglycan molecules, which played a dual role in matrix destruction. Moreover, some scholars confirmed that ADAMTS5 is also expressed in cartilage tissue and plays an important role in cartilage degradation (Santamaria et al., 2015). These studies demonstrated a strong correlation between inflammatory mediators and OA. Interestingly, our results suggested that SAA down-regulated the expression of inflammatory mediators and inhibited the catabolism of chondrocytes induced by IL-1β in a concentration-dependent manner, which was consistent with the anti-inflammatory effect of SAA in mouse macrophages (RAW264.7) reported by Huang et al. (2013).

Osteoarthritis cartilage degeneration is due to the destruction of ECM, while the synthesis and regeneration of ECM depends entirely on chondrocytes. Therefore, the survival of chondrocytes is very important for the maintenance of normal articular cartilage (Hwang and Kim, 2015). Previous studies have shown that endogenous or exogenous NO could induce chondrocyte apoptosis through a mitochondria-dependent mechanism (Wu et al., 2007). More importantly, the caspase family was closely connected with the apoptosis of eukaryotic cells. Some scholars believed that cleaved caspase-3 was the principal executor of apoptosis in the pathway of caspase-mediated apoptosis (Sharif et al., 2004). Bcl-2 family members also played an important role in the process of apoptosis. It could be divided into two categories. One was to promote cell death. For example, Bax allowed some ions and small molecules to enter the cytoplasm through the mitochondrial membrane, causing apoptosis. The other was anti-apoptosis. For example, Bcl-2 was a negative regulator of cell death and protected chondrocytes from apoptosis (Zamli and Sharif, 2011). What deserves our attention is that the anti-apoptotic effect of SAA has been found in other systems in the body. For example, SAA prevents hepatocyte apoptosis by regulating Bcl-2/Bax and caspase-3/cleaved caspase-3 signaling pathways (Wang et al., 2019). In the digestive system, SAA also protects IEC-6 cells from apoptosis by activating the Nrf2/HO-1 pathway (Zu et al., 2018). Besides, SAA relieves apoptosis and necrosis of H9c2 cells by inhibiting the ATO-induced MAPK pathway (Zhang et al., 2017). In this study, the apoptosis level of chondrocytes was evaluated by studying apoptosis-related proteins (Bax, cleaved caspase-3) as well as anti-apoptosis-related proteins (Bcl-2). We found that the results of Western blotting were consistent with those of TUNEL detection. A large number of inflammatory factors related to OA not only caused the degradation of cartilage ECM, but also led to chondrocyte inflammation-related apoptosis. Fortunately, we found that SAA significantly inhibited IL-1β-induced ECM degradation and chondrocyte apoptosis.

NF-κB is reported to be an essential transcriptional activator and is widely present in all eukaryotic cells. So far, it has been reported that NF-κB was involved in the inflammatory response, immune response, and cell death (Oeckinghaus and Ghosh, 2009). In the absence of inducer stimulation, NF-κB binds to IκBα in the cytoplasm in the form of inactivity. When stimulated by a variety of chemical and mechanical signals, IκBα was rapidly phosphorylated and degraded. Activated NF-κB enters the nucleus and triggers the expression of a large number of inflammatory factors and apoptosis-related molecules. Studies have shown that the NF-κB pathway, as the upstream of various inflammatory-related factors, plays an important role in cartilage degradation. Activation of NF-κB increases the expression of IL-1β, NO, COX-2, iNOS, and PGE2 and aggravates the destruction of articular cartilage. Previous studies have reported that *Salvia miltiorrhiza* (the source of SAA) reduces osteoarthritis-related cartilage degeneration by regulating the NF-κB signaling pathway (Xu et al., 2017). Not only that, inhibiting the activation of the NF-κB signaling pathway could also protect OA chondrocytes from apoptosis, as demonstrated by Chen et al. (Chen et al., 2018). In this study, we used the immunofluorescence method to detect the nuclear translocation of NF-κB p65 protein. The results showed that SAA inhibited the nuclear translocation of NF-κB p65, which was consistent with Western blot results. These results suggested that SAA played an anti-inflammatory and anti-apoptosis role in chondrocytes by inhibiting the activation of the NF-κB signaling pathway.

To further investigate the anti-inflammatory response of SAA in OA and the related apoptosis of chondrocytes, we studied the MAPK signaling pathway. As the main members of MAPK, ERK, p38, and JNK signaling pathways regulated many important cellular physiological and pathological processes, such as cell growth, cell differentiation, inflammatory response, and apoptosis (Plotnikov et al., 2011). Numerous studies have shown that the main biological reactions to p38 activation include the production and activation of inflammatory mediators, such as TNF-a, IL-6, and COX-2 (Hommes et al., 2003; Chung et al., 2017). Continuous activation of the JNK signaling pathway promotes apoptosis. Wang et al. reported that JNK was significantly phosphorylated during apoptosis of human SH-SY5Y cells, but apoptosis was significantly reduced after the inhibition of the JNK pathway (Wang et al., 2008). ERK is not only involved in inflammatory responses, but also involved in the regulation of many cytokines. It has been proven that inhibiting the activation of the ERK1/2 pathway helps to down-regulate the expression of catabolic factors related to the degradation of OA cartilage (Pelletier et al., 2003; Boileau et al., 2006; Saklatvala, 2007). The activation of the MAPK signaling pathway is likely to promote the inflammatory response and inflammation-related apoptosis of chondrocytes to a certain extent. However, our data indicated that SAA significantly inhibited the phosphorylation of JNK, ERK1/2, and p38. This suggests that SAA may protect chondrocytes from inflammation-related apoptosis by inhibiting the activation of the MAPK signaling pathway. In this study, we first revealed that SAA plays a protective role in IL-1β-induced inflammatory responses and related apoptosis, which may be attributed to the inhibition of the NF-κB and MAPK pathways. Unfortunately, our study does not clearly explain which of the two pathways NF-κB and MAPK played a more important role in inhibiting the inflammatory response and inflammation-related apoptosis. Therefore, the interaction of these signaling pathways in the development of OA will be further studied.

To observe the changes of articular cartilage and subcartilage bone in OA, the DMM mouse model has been used as the main model of OA *in vivo*. Pathological changes such as joint space stenosis, cartilage surface calcification, and cartilage erosion shown by the DMM mouse model were used as important evaluation indicators. In this experiment, we found that SAA could improve the pathological changes of OA and decrease the OARSI score of DMM mice through histological section staining analysis. The results of *in vitro* and *in vivo* experiments showed that the treatment of SAA alleviated the development of OA. This will also be a meaningful study as to whether prolonging the half-life of SAA *in vivo* is more beneficial to the protection of articular cartilage.

## Conclusions

In conclusion, our study finally found that SAA may play an anti-inflammatory and anti-apoptotic role in the mouse OA model by blocking the activation of the NF-κB and MAPKs (JNK, ERK1/2, and p38) pathways. This provides new insight into some of the possible mechanisms for reducing the development of OA.

## Data Availability Statement

All datasets generated for this study are included in the article/**Supplementary Material**.

## Ethics Statement

The animal study was reviewed and approved by The Animal Care and Use Committee of Wenzhou Medical University.

## Author Contributions

KY, YW, and ZW not only selected the topic, collected the relevant data, determined the research goal, and designed the experimental content but also participated in the experimental process, collected and analyzed the experimental data, and prepared the manuscripts. YW wrote this manuscript through literature search and data analysis before KY reviewed the contents of this manuscript. ZL, XF, and JZ provided advice and assistance for the experimental process, data collection, and analysis. The content of the manuscript was approved by all authors.

## Funding

This study was supported by grants from Zhejiang Province Science and Technology Department Public Welfare Technology Research Program/Experimental Animal Project (LGD19H060001), Zhejiang Province Chinese Medicine Science and Technology Project (2013ZQ024), and the Zhejiang Province Health Department Project (2019RC052).

## Conflict of Interest

The authors declare that the research was conducted in the absence of any commercial or financial relationships that could be construed as a potential conflict of interest.

## Supplementary Material

The Supplementary Material for this article can be found online at: https://www.frontiersin.org/articles/10.3389/fphar.2020.00682/full#supplementary-material
