# Supplementary figures and images for: Salvianolic Acid A Has Anti-Osteoarthritis Effect In Vitro and In Vivo
Source: Front Pharmacol. 2020 Jun 3;11:682. doi: 10.3389/fphar.2020.00682 (PMC7283387; doi:10.3389/fphar.2020.00682)

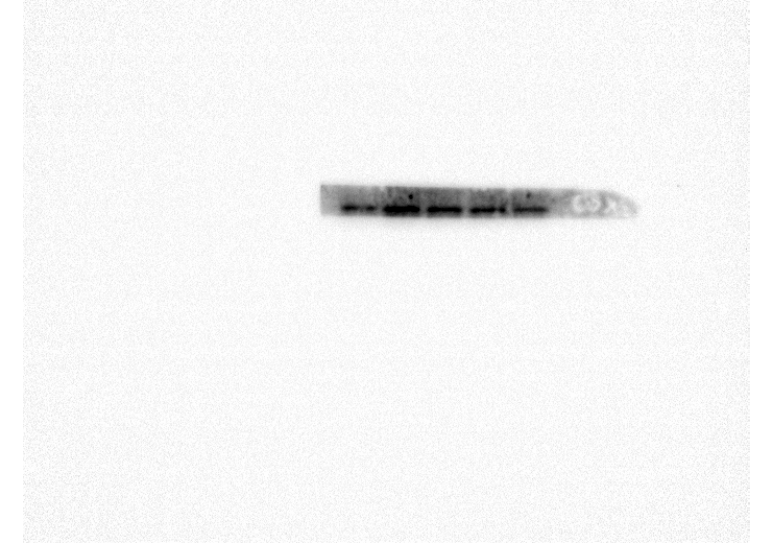

Supplement: Supplementary file 1 [file DataSheet_1.zip › Supplement-ary material/Figure 3/COX-2 (Figure 3).tif]

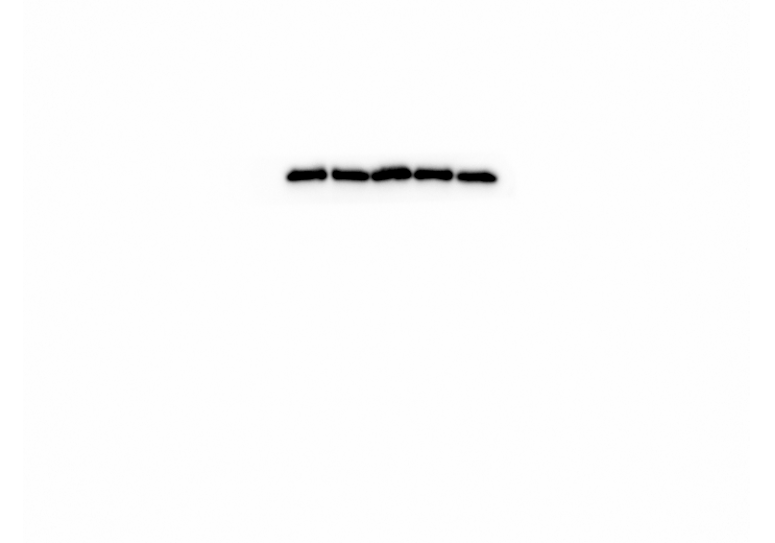

Supplement: Supplementary file 1 [file DataSheet_1.zip › Supplement-ary material/Figure 3/GADPH (Figure 3).tif]

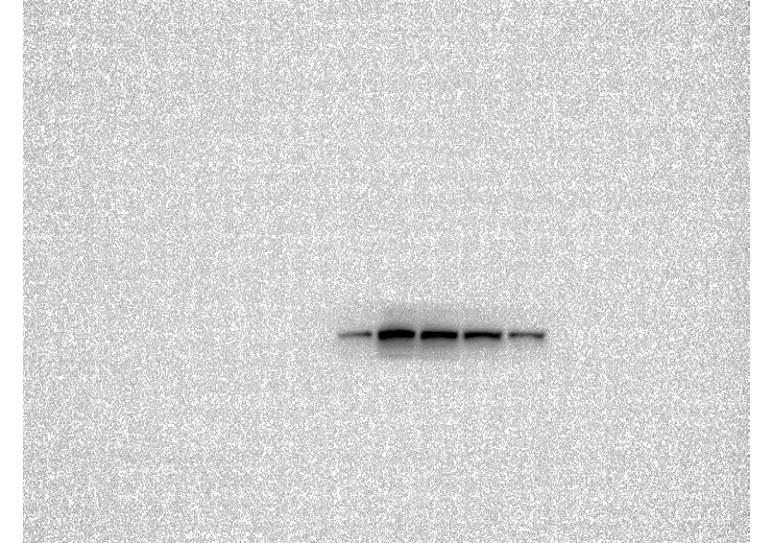

Supplement: Supplementary file 1 [file DataSheet_1.zip › Supplement-ary material/Figure 3/INOS (Figure 3).tif]

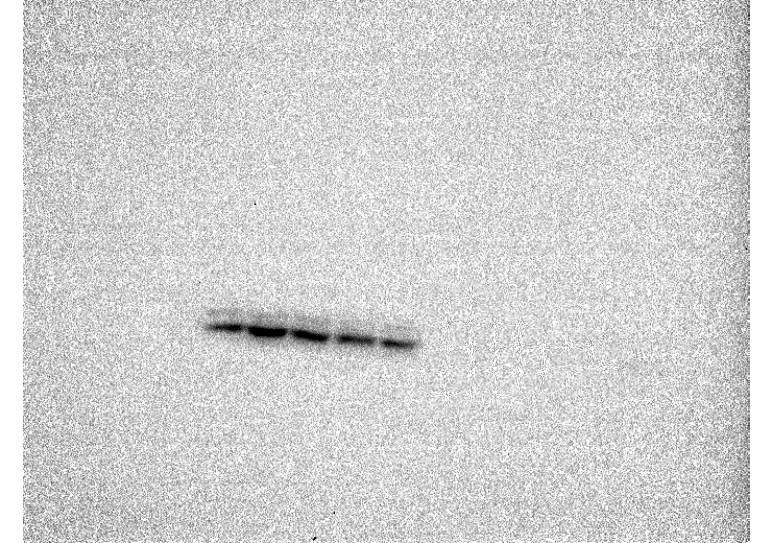

Supplement: Supplementary file 1 [file DataSheet_1.zip › Supplement-ary material/Figure 4/ADAMTS5 (Figure 4).tif]

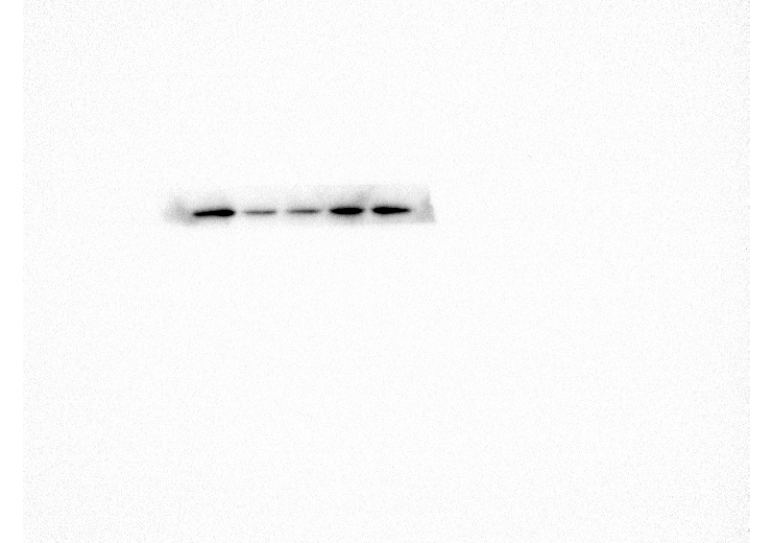

Supplement: Supplementary file 1 [file DataSheet_1.zip › Supplement-ary material/Figure 4/Collagen II (Figure 4 ).tif]

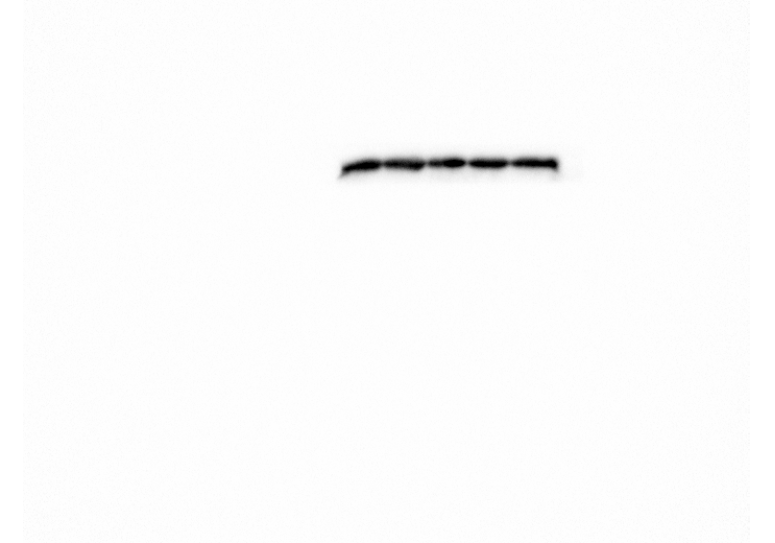

Supplement: Supplementary file 1 [file DataSheet_1.zip › Supplement-ary material/Figure 4/GADPH (Figure 4).tif]

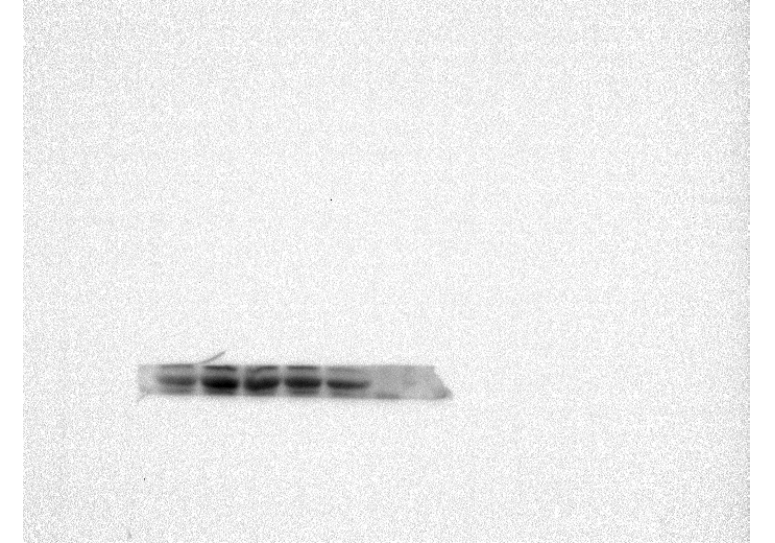

Supplement: Supplementary file 1 [file DataSheet_1.zip › Supplement-ary material/Figure 4/MMP3 (Figure 4).tif]

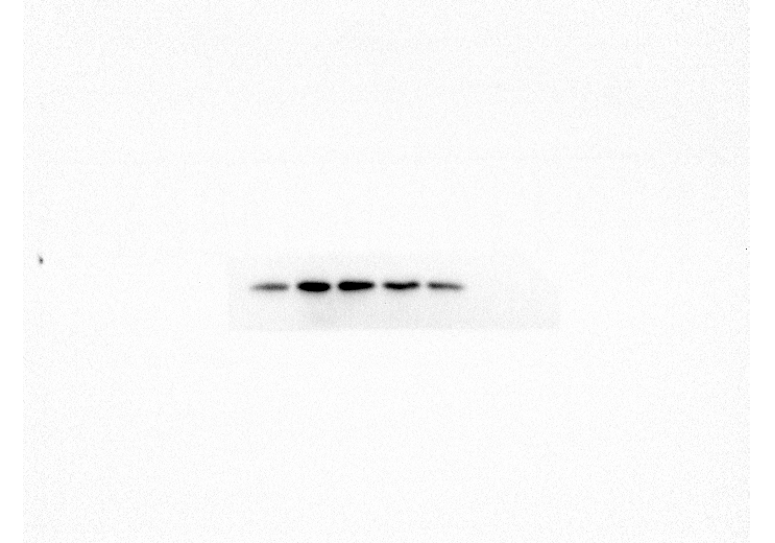

Supplement: Supplementary file 1 [file DataSheet_1.zip › Supplement-ary material/Figure 6/Bax (Figure 6).tif]

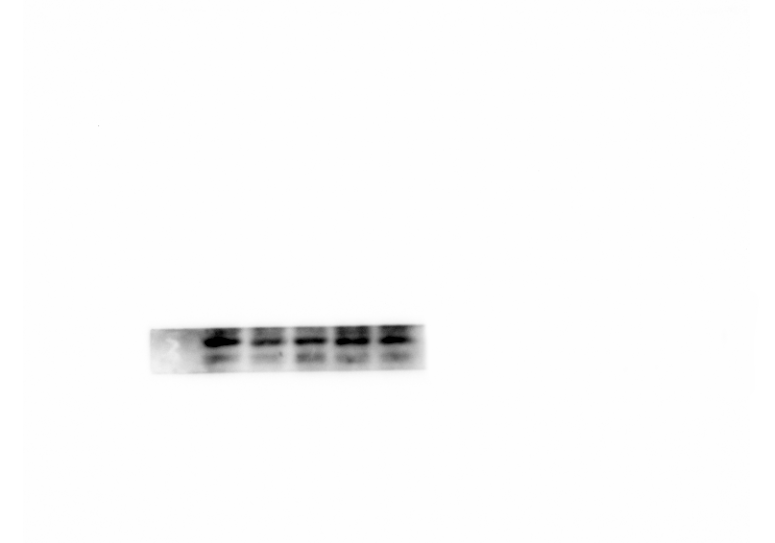

Supplement: Supplementary file 1 [file DataSheet_1.zip › Supplement-ary material/Figure 6/Bcl-2 (Figure 6).tif]

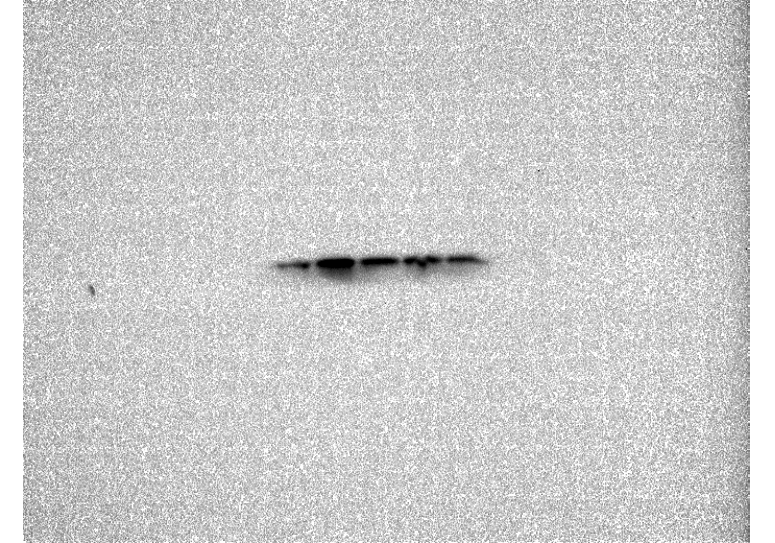

Supplement: Supplementary file 1 [file DataSheet_1.zip › Supplement-ary material/Figure 6/Cleaved-caspase 3 (Figure 6).tif]

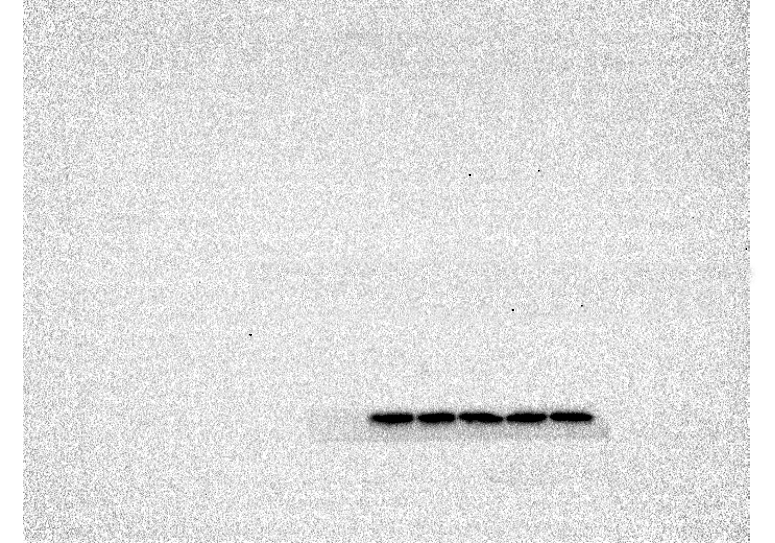

Supplement: Supplementary file 1 [file DataSheet_1.zip › Supplement-ary material/Figure 6/GADPH (Figure 6).tif]

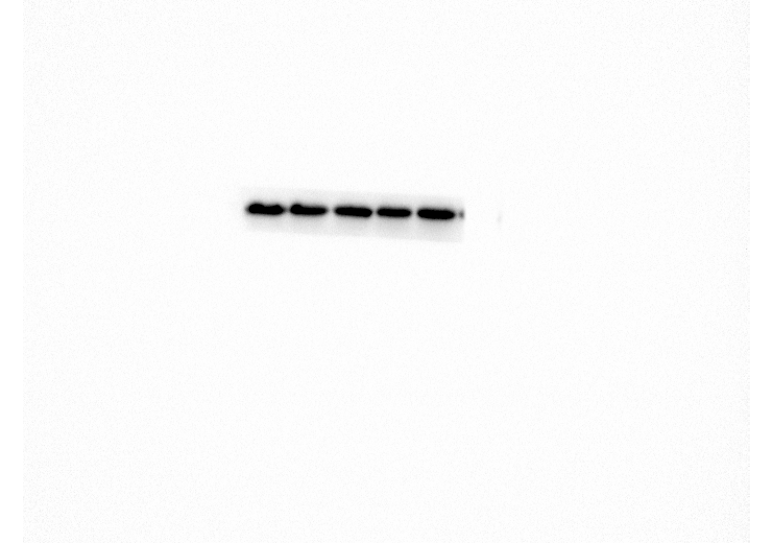

Supplement: Supplementary file 1 [file DataSheet_1.zip › Supplement-ary material/Figure 7/GADPH (Figure 7).tif]

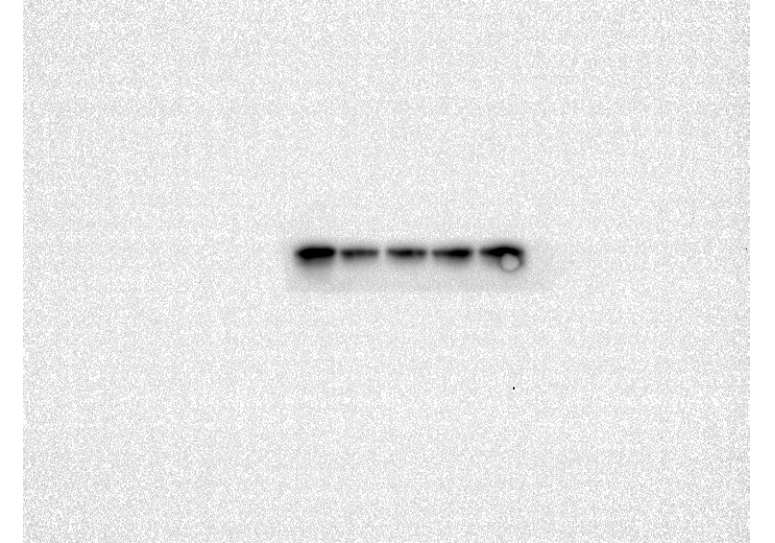

Supplement: Supplementary file 1 [file DataSheet_1.zip › Supplement-ary material/Figure 7/Ia╩Ba┴ (Figure 7).tif]

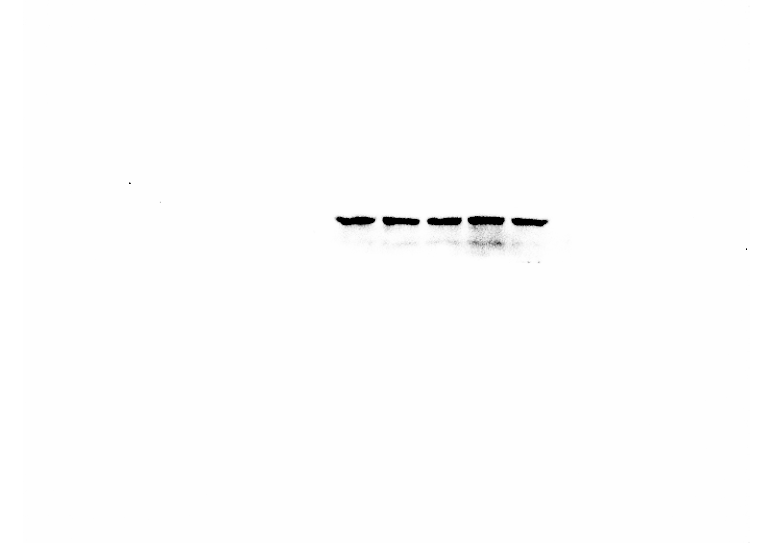

Supplement: Supplementary file 1 [file DataSheet_1.zip › Supplement-ary material/Figure 7/Lamin B1 (Figure 7).tif]

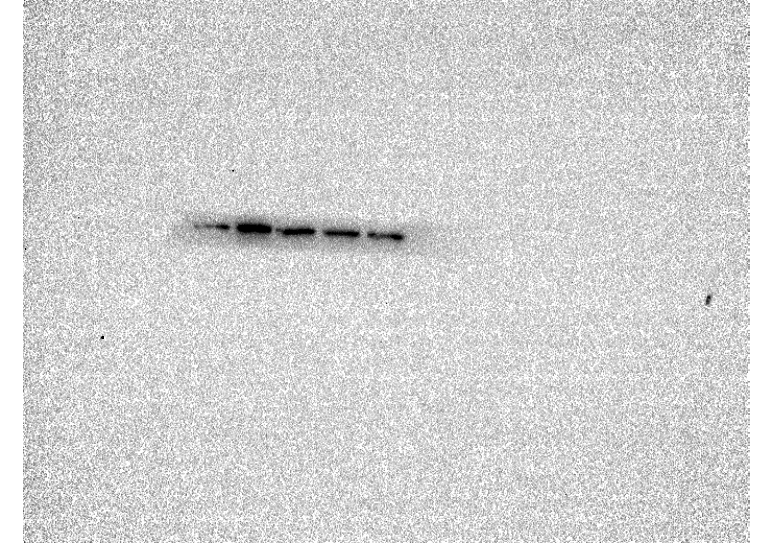

Supplement: Supplementary file 1 [file DataSheet_1.zip › Supplement-ary material/Figure 7/P65 (Figure 7).tif]

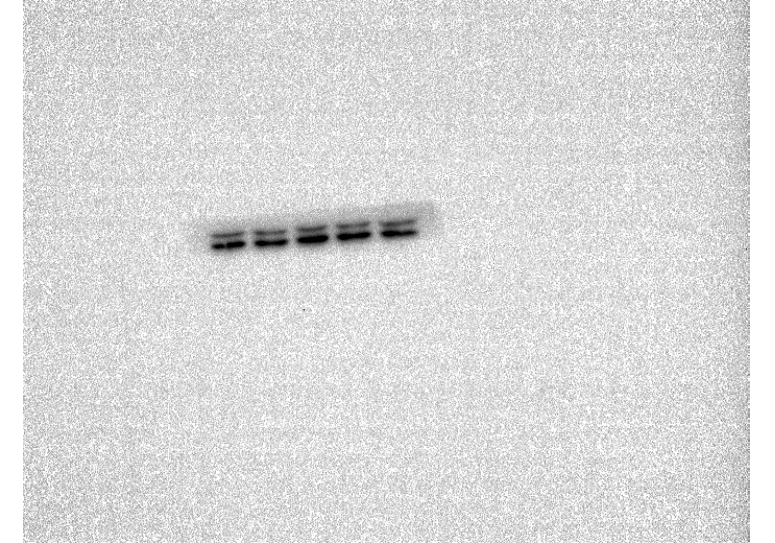

Supplement: Supplementary file 1 [file DataSheet_1.zip › Supplement-ary material/Figure 9/ERKú¿Figure 9ú⌐.tif]

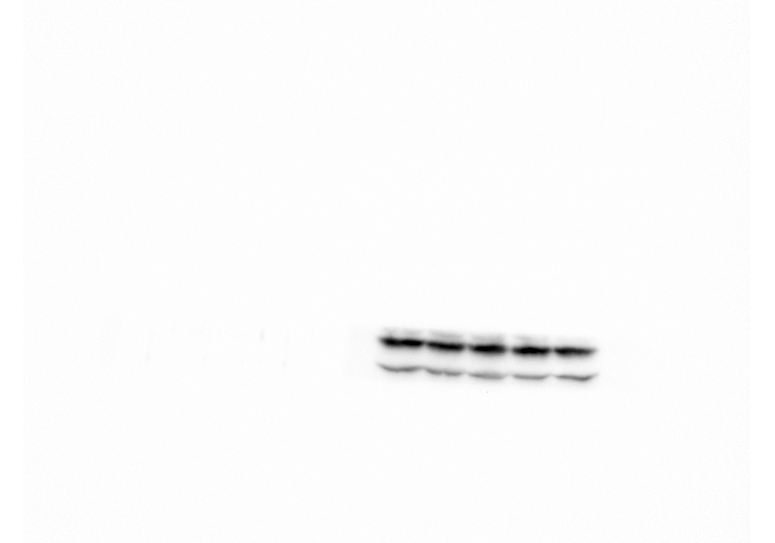

Supplement: Supplementary file 1 [file DataSheet_1.zip › Supplement-ary material/Figure 9/JNKú¿Figure 9).tif]

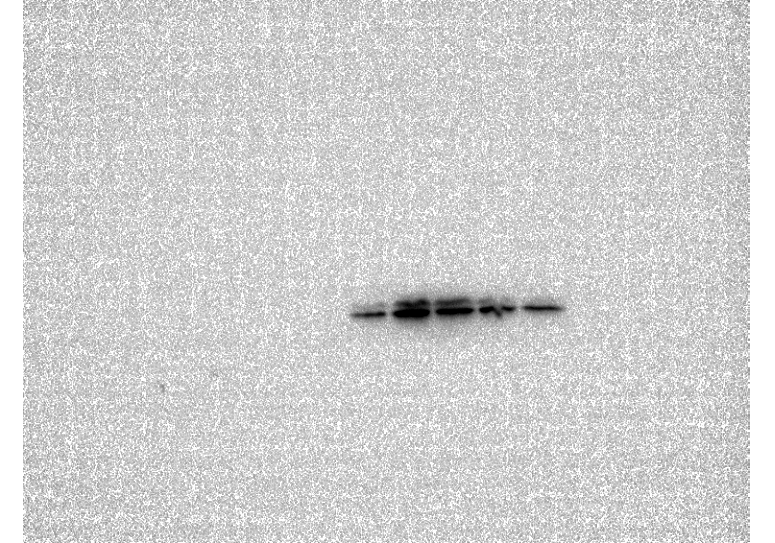

Supplement: Supplementary file 1 [file DataSheet_1.zip › Supplement-ary material/Figure 9/P-ERK (Figure 9ú⌐.tif]

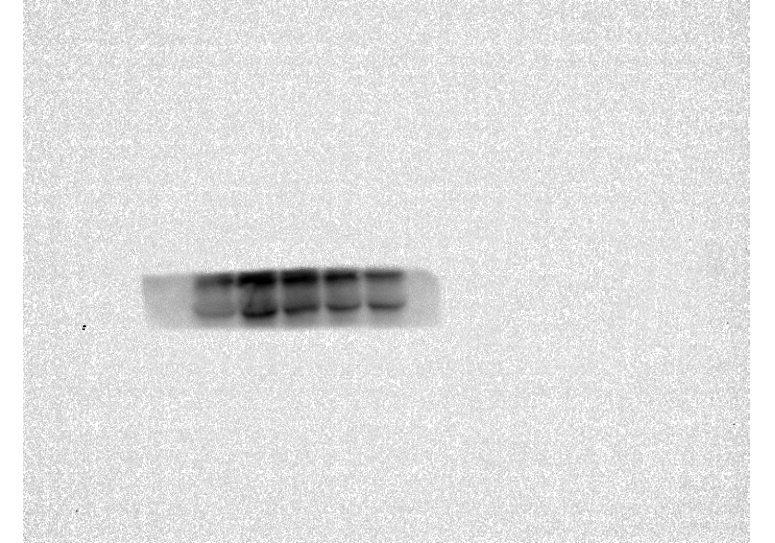

Supplement: Supplementary file 1 [file DataSheet_1.zip › Supplement-ary material/Figure 9/P-JNK (Figure 9ú⌐.tif]

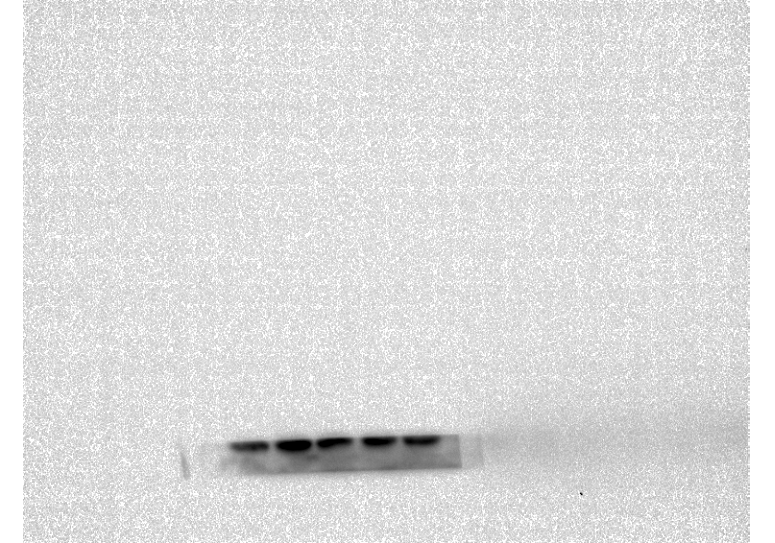

Supplement: Supplementary file 1 [file DataSheet_1.zip › Supplement-ary material/Figure 9/p-p38ú¿Figure 9).tif]

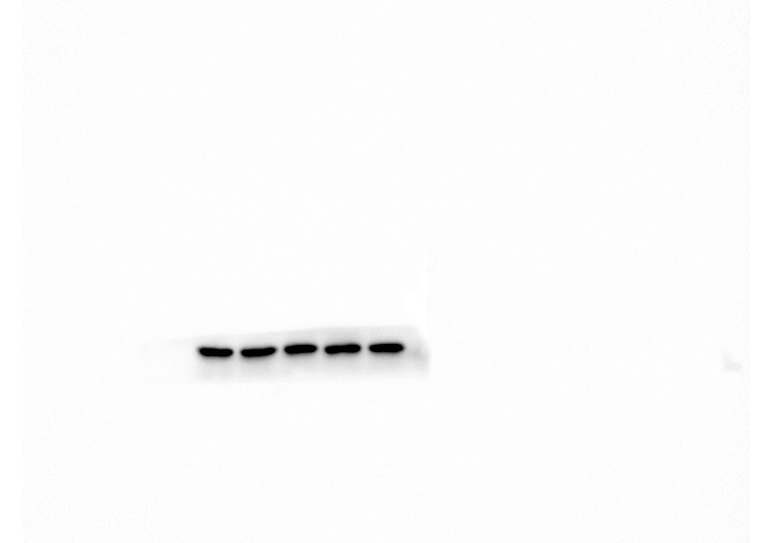

Supplement: Supplementary file 1 [file DataSheet_1.zip › Supplement-ary material/Figure 9/p38ú¿Figure 9ú⌐.tif]
